# The synergistic effect of Canady Helios cold atmospheric plasma and a FOLFIRINOX regimen for the treatment of cholangiocarcinoma in vitro

**DOI:** 10.1038/s41598-021-88451-w

**Published:** 2021-04-26

**Authors:** Olivia Jones, Xiaoqian Cheng, Saravana R. K. Murthy, Lawan Ly, Taisen Zhuang, Giacomo Basadonna, Michael Keidar, Jerome Canady

**Affiliations:** 1 Jerome Canady Research Institute for Advanced Biological and Technological Sciences, Takoma Park, MD 20912 USA; 2 Plasma Medicine Life Sciences, Takoma Park, MD 20912 USA; 3 Department of Surgery, University of Massachusetts School of Medicine, Worcester, MA 01655 USA; 4 School of Engineering and Applied Science, The George Washington University, Washington, DC 20052 USA

**Keywords:** Bile duct cancer, Bile duct cancer, Cancer therapy

## Abstract

Cholangiocarcinoma (CCA) is a rare biliary tract cancer with a low five-year survival rate and high recurrence rate after surgical resection. Currently treatment approaches include systemic chemotherapeutics such as FOLFIRINOX, a chemotherapy regimen is a possible treatment for severe CCA cases. A limitation of this chemotherapy regimen is its toxicity to patients and adverse events. There exists a need for therapies to alleviate the toxicity of a FOLFIRINOX regimen while enhancing or not altering its anticancer properties. Cold atmospheric plasma (CAP) is a technology with a promising future as a selective cancer treatment. It is critical to know the potential interactions between CAP and adjuvant chemotherapeutics. In this study the aim is to characterize the efficacy of FOLFIRINOX and CAP in combination to understand potential synergetic effect on CCA cells. FOLFIRINOX treatment alone at the highest dose tested (53.8 µM fluorouracil, 13.7 µM Leucovorin, 5.1 µM Irinotecan, and 3.7 µM Oxaliplatin) reduced CCA cell viability to below 20% while CAP treatment alone for 7 min reduced viability to 3% (p < 0.05). An analysis of cell viability, proliferation, and cell cycle demonstrated that CAP in combination with FOLFIRINOX is more effective than either treatment alone at a lower FOLFIRINOX dose of 6.7 µM fluorouracil, 1.7 µM leucovorin, 0.6 µM irinotecan, and 0.5 µM oxaliplatin and a shorter CAP treatment of 1, 3, or 5 min. In conclusion, CAP has the potential to reduce the toxicity burden of FOLFIRINOX and warrants further investigation as an adjuvant therapy.

## Introduction

Cholangiocarcinoma (CCA) is a rare and aggressive malignancy arising in the intrahepatic or extrahepatic biliary tract. It is often discovered in advanced late stages, and the prognosis is poor with a five-year survival rate under 20%^1,2^. Surgical resection or liver transplantation at an early stage are the best option for curative treatment of CCA^3^. However, CCA has a high recurrence rate after surgical resection^4^. Chemoresistance also presents a challenge in administering adjuvant chemotherapy and as a result, CCA is known for poor clinical outcomes^5,6^.

For patients with recurrent CCA, gemcitabine and fluorouracil (5-FU) have been standard options as individual treatments or drug combination therapy for years^7–9^. The FOLFIRINOX protocol is a drug regimen consisting of fluorouracil (5-FU), leucovorin, irinotecan, and oxaliplatin. The regimen is not yet standard clinical practice for CCA. In a phase two–three clinical trial, FOLFIRINOX increased overall survival over gemcitabine treatment in patients with metastatic pancreatic cancer^10^. However the regimen was not well tolerated; the incidence of thrombocytopenia, neutropenia, and febrile neutropenia were significantly higher in FOLFIRINOX treatment patients^10^. To address this limitation, studies have focused on reducing dose or modifying the four components. Dosage iterations of modified FOLFOX-4, FOLFOX-5, and FOLFOX-7 have been used to treat pancreatic, colorectal, and bladder cancers^11–13^.

Cold atmospheric plasma (CAP) has been extensively studied in various biomedical fields. It is a novel approach to targeted cancer treatment and has demonstrated its anti-cancer effects in vitro^14–17^. The detailed mechanism have not been fully elucidated, however studies have established that CAP selectively induces apoptosis and DNA damage in tumor cells^18–20^. Further research indicates low doses of CAP does not damage normal tissue^21–24^. Recently, indirect CAP treatment was effective for the treatment of CCA in vitro*,* selectively killing CCA cells over normal hepatocytes^25^. Research on CAP in combination with other therapies has shown some potential synergism with anti-neoplastic agents in melanoma cells^26^, drug loaded nanoparticles in breast cancer cells^27^, gemcitabine in murine pancreatic cancer cells^28^, and temozolomide in glioblastoma cells^29^.

The Canady Helios Cold Plasma System (CHCPS) paired with the Canady Helios Cold Plasma Scalpel has potential as an anti-cancer therapy (U.S. Patent No. 9999462)^30^. The CHCPS is currently subject to a phase I FDA Investigational Device Exemption Approval clinical trial in the United States and Israel. In this trial the Canady Helios Cold Plasma Scalpel delivers cold plasma at the surgical margins immediately after tumor resection. The temperature of our device during use ranges between 26 and 31 °C^20^. The CHCPS reduces viability of solid tumor cells and does not thermally damage normal tissue^14,31^. The system has shown efficacy in breast cancers representative of four molecular subtypes, and a 92–99% reduction in viability was achieved 48 h after CAP treatment (p < 0.05)^32^.

There is a need for investigation into therapies for CCA due to its poor prognosis, chemoresistance, and high recurrence rate. CAP is a potential adjuvant treatment. In this in vitro study dose-dependent experiments were performed on the human intrahepatic cholangiocarcinoma (ICCA) cell line (KKU-055) to establish efficacy of CAP and FOLFIRINOX in combination. Various dose levels of both therapies individually and in combination were used to quantify changes in cell viability and cell cycle progression. FOLFIRINOX was administered as a first line therapy followed by CAP treatment to combine anti-cancer effects of both. We are the first to demonstrate the in vitro synergistic effects of FOLFIRINOX and CAP.

## Materials and methods

### Cell culture

The intrahepatic poorly differentiated cholangiocarcinoma cell line, KKU-055, was purchased from Sekisui XenoTech, LLC (Kansas City, KS). Cells were cultured in Dulbecco’s Modified Eagle Medium (DMEM) supplemented with 10% fetal bovine serum and 1% Pen Strep (Thermo Fisher Scientific, Waltham, MA, USA). Cells were lifted with Trypsin–EDTA and seeded in 12-well plates at 100,000 cells/well or 50,000 cells/well in 1 mL complete media. Cells were then incubated 24 h at 37 °C and 5% CO_2_ prior to drug or CAP treatment. All experiments were performed at the Jerome Canady Research Institution for Advanced Biological and Technological Sciences (JCRI-ABTS) in Takoma Park, MD, USA.

### FOLFIRINOX treatment

The four FOLFIRINOX drugs were individually diluted in dimethyl sulfoxide DMSO then combined in a stock solution at the clinical dose ratio of oxaliplatin (Sigma Aldrich #PHR1528) 85 mg/m^2^, leucovorin (Sigma Aldrich #PHR1541) 400 mg/m^2^, irinotecan (Sigma Aldrich #I1406) 180 mg/m^2^, and 5-fluorouracil (Sigma Aldrich #PHR1227) 400 mg/ m^2^. In patient dose calculations the initial 400 mg/ m^2^ 5-FU bolus is followed by an infusion of more 5-FU over 46 h. For the purpose of this study the bolus 5-FU dosage was given to treat cells at a singular timepoint. FOLFIRINOX doses will be referred to by their corresponding concentration of 5-fluorouracil [5-FU]. Further dilutions of the four drugs into the FOLFIRINOX mix were made with complete cell culture media. After cells had been seeded and incubated for 24 h, the FOLFIRINOX dosage of choice was added to each well. Cells were then incubated for 24 h with the drug before further treatment.

FOLFIRINOX dosage was calculated in the following manner. The clinical dose given in mg/m^2^ was converted mg/cm^2^ to correspond with the surface area of one well in a 12-well plate. The four drugs were diluted with DMSO to get mmol/mL that corresponded with the clinical recommendation. Then this drug stock solution was diluted 1:10 with complete DMEM media and finally 1:1000 when treating cells in a 12-well plate to a final µM concentration of each drug.

### Cold plasma device

All CAP treatments were generated with a US Medical Innovations LLC 22–601 MCa high frequency electrosurgical generator, a Canady Helios Cold Plasma System, paired with a Canady Helios Cold Plasma Scalpel^30^. All CCPCS tests were conducted with a constant helium flow rate of 3 L/min, at a power setting of 120p which corresponds to 28.7 W^14^. Treatment durations were up to 7 min. The distance between the tip of the plasma scalpel and media surface was constant at 1.5 cm. Immediately after CAP treatment, cells were transferred to a 37 °C and 5% CO_2_ humidified incubator and cultured up to 72 h. CAP treatments were done 48 h after initial cell seeding. Drug treated cells had finished a 24 h incubation with FOLFIRINOX, and CAP only or non-treated cells remained in the incubator the entire time prior to CAP.

### Cell viability assay

Cellular viability and proliferation were assessed through a Thiazolyl Blue Tetrazolium Bromide (MTT, Abcam ab146345) assay performed 48 h after CAP treatment. Cells were incubated with MTT solution for 3 h at 37 and 5% CO_2_ humidified incubator. The absorbance of the dissolved compound was measured by BioTek Synergy HTX (Winooski, VT, USA) microplate reader at 570 nm. Viability assays were repeated at least 3 times with a minimum of 2 intra experimental replicates. For each assay cell viability was calculated by normalizing to non-treated cells.

### Confocal microscopy and Ki67 staining

Confocal microscopy analysis was prepared in the following manner. One round platinum lined cover glass 12 mm in diameter was placed in each well of a 12-well plate then coated with fibronectin and collagen II for at least 12 h. Cells were then seeded on cover glass inside of wells to normalize treatment to MTT assays and IncuCyte analysis. After selected drug treatment, CAP treatment, or combination treatment cultures were fixed with ice cold (− 20 °C) methanol for 10 min. Then cells were stained with Alexa Fluor 488-conjugated Ki-67 Rabbit mAb (Cell Signaling Technology, #11882) or Isotype control (Cell Signaling Technology, #4340) antibodies according to Immunofluorescence General Protocol by Cell Signaling Technology (Danvers, MA, USA). Cells were incubated overnight at 4 °C protected from light. The cover slides were then carefully moved onto glass slides and covered with Antifade Mounting Reagent with DAPI (Vector Laboratories, H-1500) drops and then a 1 mm cover slide. Slides were allowed to cure for up to 2 nights in a 4 °C refrigerator then sealed with clear nail polish. Images were taken with Zeiss Confocal 510 LSM (Oberkochen, Germany), analyzed with Zeiss ZenLite (2012) software, and Ki-67 positivity was calculated in Microsoft Excel 2019 (Redmond, WA, USA).

### Cell cycle

Cell cycle phase contrast images were collected on the IncuCyte Live-Cell Analysis System (Essen Bioscience, Ann Arbor, MI). A stable KKU-055 cell line was established through 5 μg/mL puromycin (Sigma Aldrich P8833) selection after transfection with the IncuCyte Red/Green Lentivirus Reagent (IncuCyte #4779) for labeling and indication of in vitro cell cycle. Red indicated G1 phase and Green indicated S/G2/M phases while unlabeled cells indicated M-G1 transition phase or dead cells. In-vitro cell growth images were collected at 1-h intervals up to 72 h after each treatment condition. The percent of cell confluence and detailed cell counts per well were quantified by the IncuCyte Cell By Cell Analysis then plotted in Microsoft Excel 2019.

### Statistics

Data was plotted by Microsoft Excel 2019 as mean ± standard error of the mean. Student unpaired t-tests and two-way analysis of variance (ANOVA) were used to determine significant differences between the groups. Significant CAP-drug combination effects were followed by post hoc tests with Bonferroni correction. To determine significance of independent and combined treatment groups with *p-value* < 0.05 considered statistically significant.

## Results

### FOLFIRINOX regimen reduced cholangiocarcinoma cell viability

**Figure 1:**
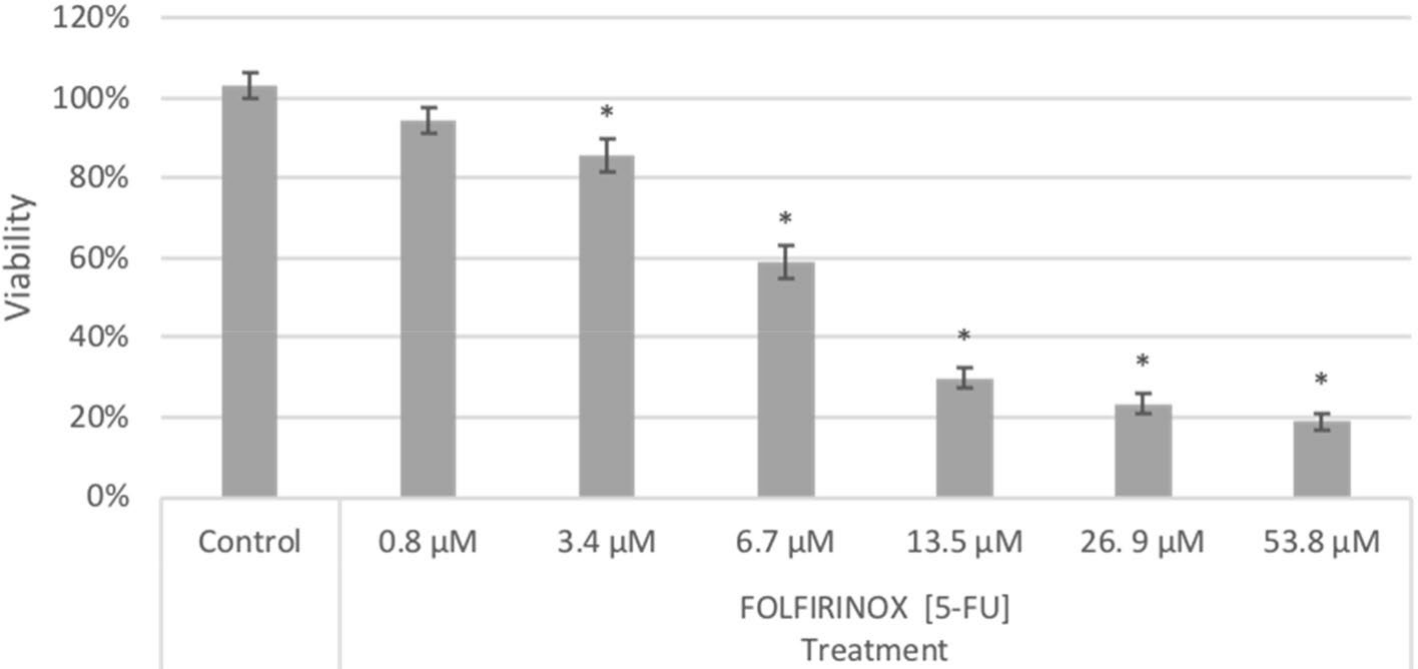
Reduction of KKU-055 cell viability after 48-h exposure to FOLFIRINOX, compared to DMSO treated cells controls (mean ± SEM). Each drug dosage level is labeled by the corresponding concentration of 5-fluorouracil (cohort = 4, 2 /cohort, n = 8, *t* test). *p < 0.05.

To determine the possible synergistic effects of FOLFIRINOX on KKU-055 cells, an optimal dosage of the four drugs in combination must be able to reduce cell viability significantly. A serial dilution of 6 doses of FOLFIRINOX was done to establish a baseline toxicity measurement for each dose (Table 1 and Fig. 1). Control cells treated with DMSO remained viable 99% (± 6) suggesting that DMSO had no significant effects on cell growth, and all reduction was due to FOLFIRINOX (cohort = 4, 2/cohort, n = 8, *t* test p > 0.05). Cell viability decreased significantly at doses equal to or higher than the 3.4 µM 5-FU level (cohort = 4, 2/cohort, n = 8, *t* test p < 0.05). Exposure to the lowest FOLFIRINOX dose decreased viability to 94% (± 3) and was not statistically significant compared to the control DMSO which reached 103% (± 3). When KKU-055 cells were treated with the highest dose of FOLFIRINOX (53.8 µM Fluorouracil, 13.1 µM Leucovorin, 5.1 µM Irinotecan, and 3.7 µM Oxaliplatin), cell viability was reduced to 19% (± 1.9).

**Table 1:** Drug concentrations of the four FOLFIRINOX components for each dose level in the serial dilution.

Drug	Dose 1	Dose 2	Dose 3	Dose 4	Dose 5	Dose 6
5-Fluorouracil (5-FU)	0.8	3.4	6.7	13.5	26.9	53.8
Leucovorin	0.2	0.9	1.7	3.4	6.8	13.7
Irinotecan	0.1	0.3	0.6	1.3	2.5	5.1
Oxaliplatin	0.1	0.2	0.5	0.9	1.9	3.7

This corresponds to the [5-FU] notation in Fig. 1.

### Assessment of the combined treatment of CAP and FOLFIRINOX

**Figure 2:**
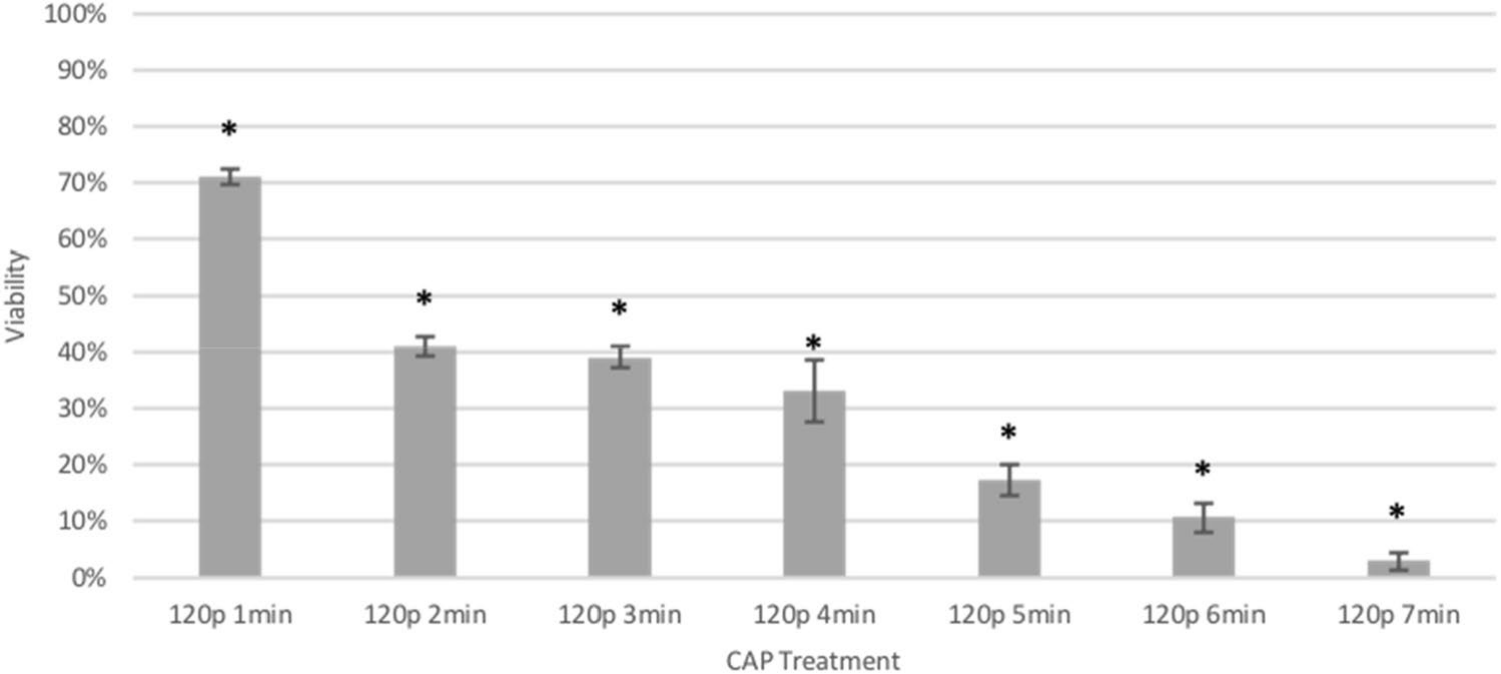
Reduction of KKU-055 cell viability 48 h after CAP treatment for 1–7 min at 120p which corresponds to 28.7 W compared to untreated controls (cohort = 4, 2 /cohort, n = 8 *t* test). *p < 0.05.

A dose dependence experiment was performed on KKU-055 cells to establish CAP efficacy. MTT assays were conducted 48 h post CAP treatment. Cell viability was significantly reduced by CAP for all durations, and the highest treatment of 7 min reduced viability to 3% (p < 0.005, Fig. 2).

**Figure 3:**
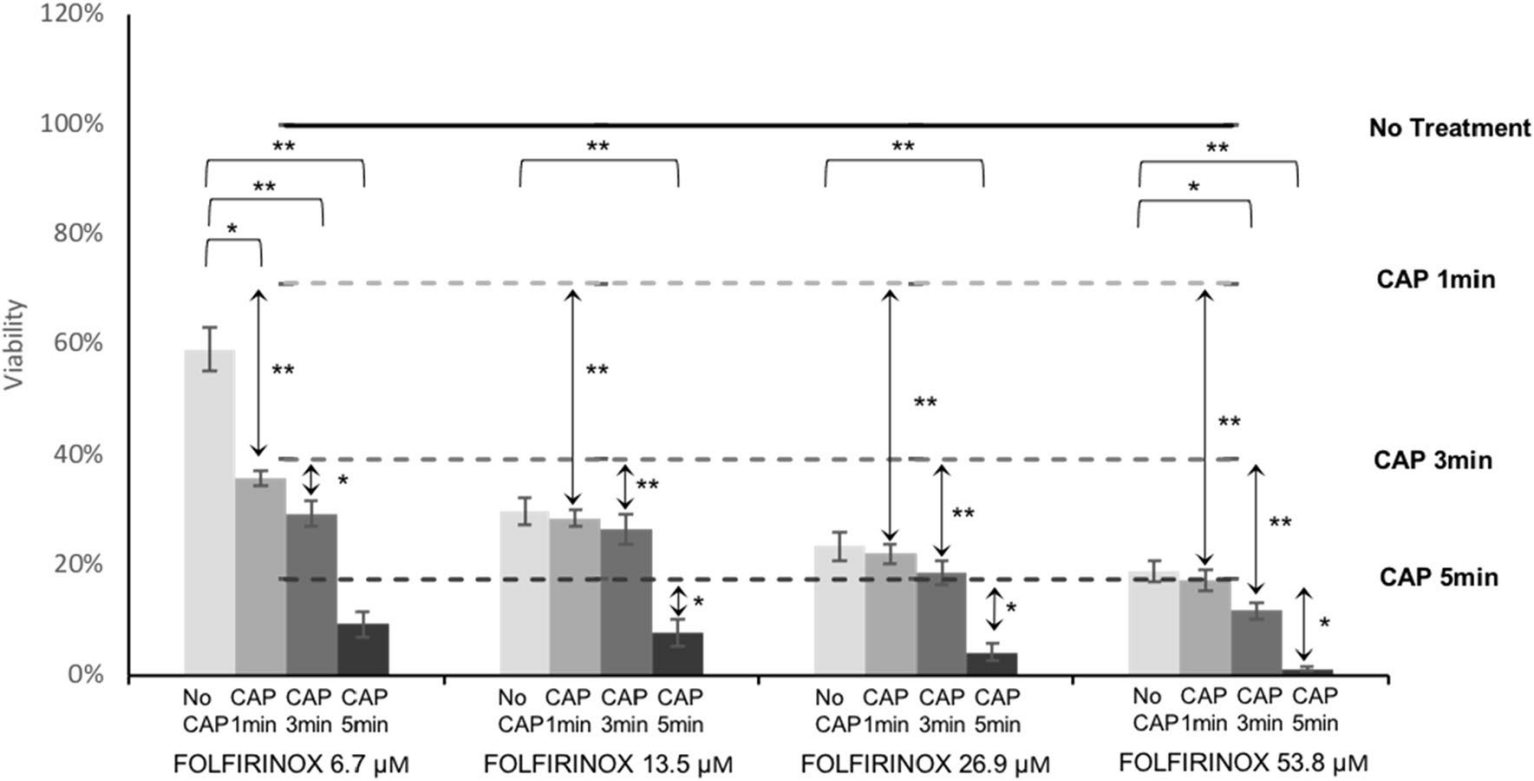
The effect of adjunctive FOLFIRINOX treatment in combination with CAP on cholangiocarcinoma cell viability. Four drug dosages, labeled by their corresponding concentration of 5-fluorouracil (5-FU) from Table 1, were combined with three CAP doses of either 1, 3 or 5 min. FOLFIRINOX treated cells were subject to 24 h pretreatment incubation before CAP, and MTT assays were performed 48 h after CAP treatment. *T* tests were used to determine synergetic treatment combinations and are indicated as *p < 0.05 or **p < 0.005.

KKU-055 cells were exposed to 24 h of FOLFIRINOX pretreatment at 6.7–53.8 µM [5-FU] (Table 1) and CAP at 120p for 1, 3, or 5 min. Viability reduction was measured 48 h after treatment (Fig. 3). Cells without either treatment were negative controls. Complete cell death was observed with a combination of FOLFIRINOX (53.8 µM 5-FU dose) and CAP for 5minutes where viability was reduced to 1%.

A two-way ANOVA test followed by post hoc Fisher exact tests (with Bonferroni correction) was conducted on this combination treatment experiment. Sources of variation were a change in either CAP dose or FOLFIRINOX dose. Then the variance between the two was tested to determine if one treatment had an effect of the other. There were three hypotheses for this test; H_1_: The observed viability between drug dosage groups is equal, H_2_: the observed viability between CAP dosage groups is equal, and H_3_: there is no interaction between the two treatments. For all three hypotheses p < 0.05, so we can reject each one. Student paired t-tests and two-way ANOVA test followed by post hoc Fisher exact tests (with Bonferroni correction) were then conducted to compare each combination treatment with every other experiment group (Table 2).

**Table 2:**
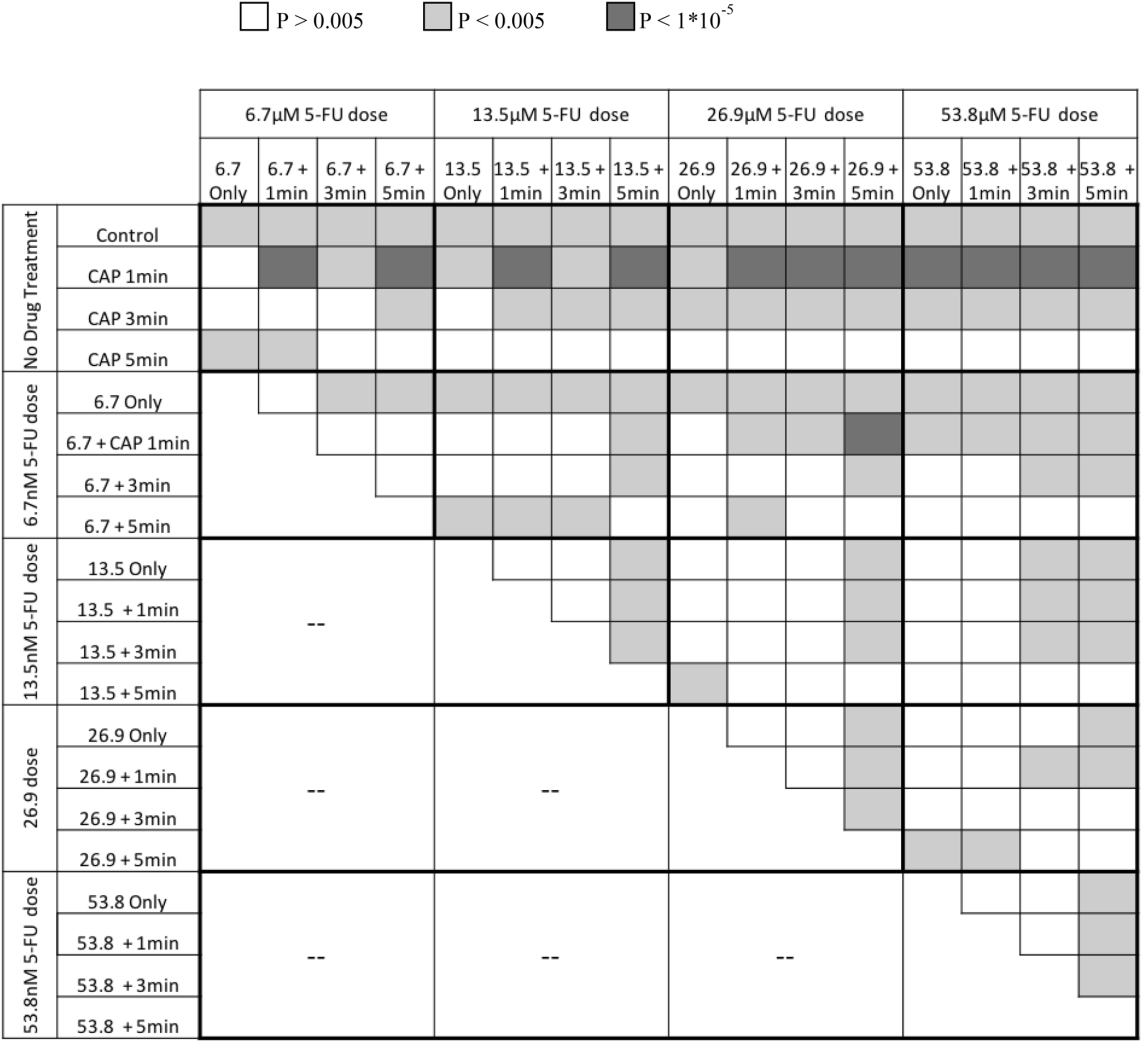
Chart showing the comparison of the reduction of viability between treatment groups.

Whether there is statistical difference p < 0.005 and if that difference is extremely significant p < 1 × 10^–5^ (Student’s t test with Bonferroni’s correction).

Dosage combinations were considered synergetic when combination treatment reduced viability significantly more than the corresponding CAP or FOLFIRINOX dosage alone. In cases when the FOLFIRINOX dose was 13.5 µM [5-FU] or higher the drug alone was strong enough to reduce KKU-055 viability to below 30%, and this made drug treatment significantly more effective than 1 or 3 min of CAP (cohort = 4, 2/cohort, n = 8 *t* test p < 0.05, Fig. 3, Table 2). With these high doses of drug enhanced efficacy then could not be determined. Statistically significant for all the drug combinations are indicated in Table 2.

The FOLFIRINOX dose (6.7 µM fluorouracil, 1.7 µM leucovorin, 0.6 µM irinotecan, and 0.5 µM oxaliplatin) in combination with 5 min of CAP achieved a 91% reduction in cell viability (Fig. 3). This FOLFIRINOX dose in combination with 5 min of CAP was more effective in reducing cell viability than the drug alone (p < 0.001). Also, when this dose was combined with 1 min of CAP the combination treatment was statistically more effective than 1 min of CAP alone (p < 1 × 10^–5^). The efficacy of this FOLFIRINOX dose in combination treatments was observed and the drug alone did not statistically reduce cell viability more than CAP alone so this dosage was selected for following confocal microscopy and cell cycle analysis.

### Decrease in cell proliferation

**Figure 4:**
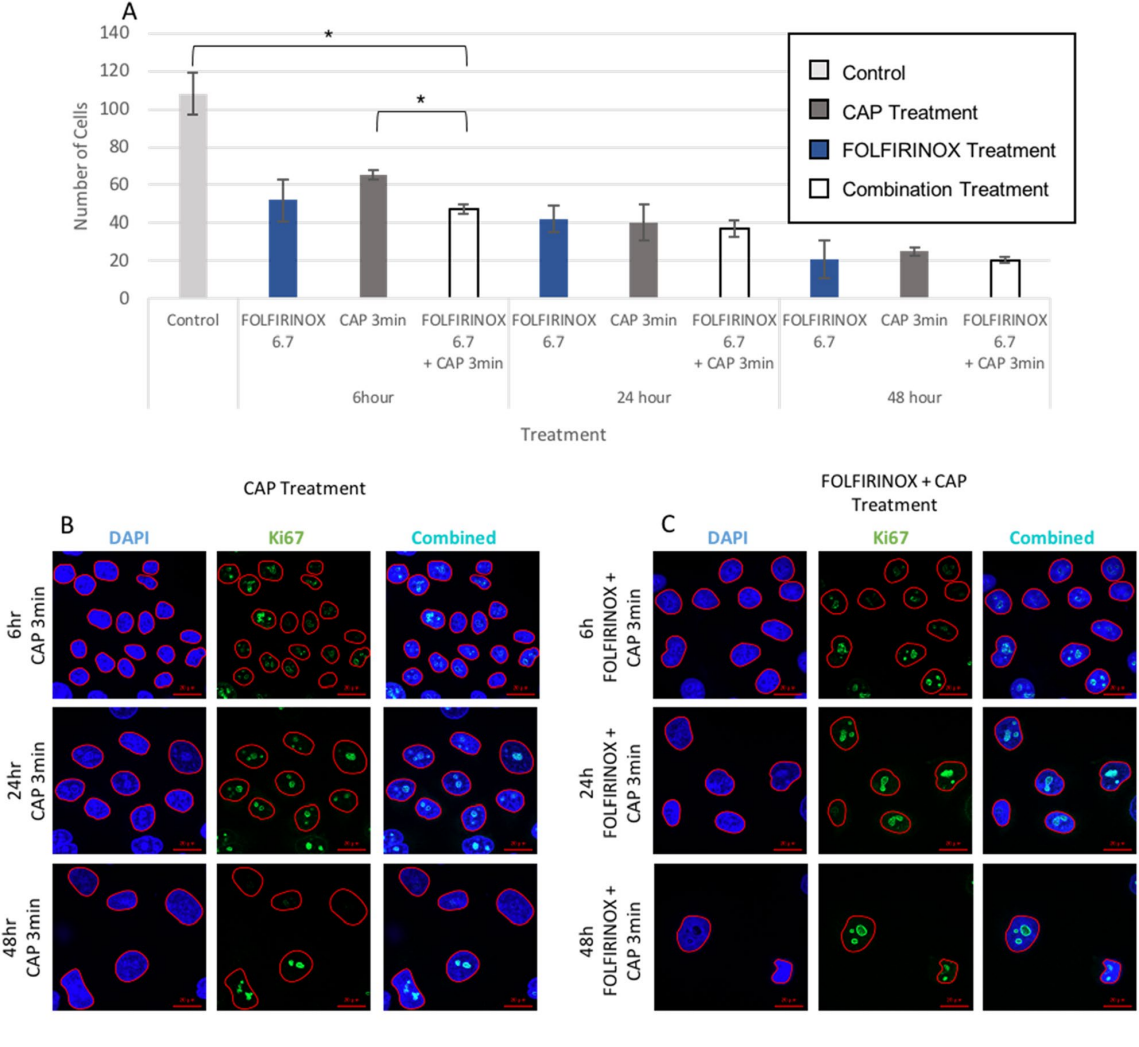
KKU-055 cells were imaged 6, 24, and 48 h after CAP or CAP and FOLFIRINOX treatments with an untreated negative control. (**A**) The total number of cells in five representative images per treatment condition is plotted (cohort = 3, 2/cohort, n = 6, *t* test). *p < 0.05. (**B**) Representative images of Ki67 and DAPI staining of cells after CAP treatment at 120p for 3 min. (**C**) Representative images of cells subject to 24 h pretreatment with FOLFIRINOX (6.7 µM 5-FU, 1.7 µM leucovorin, 0.6 µM irinotecan, and 0.5 µM oxaliplatin) before CAP at 120p for 3 min.

Cell proliferation was examined by Ki-67/DAPI co-staining at 6, 24, or 48 h post CAP, FOLFIRINOX, or combination treatment*.* The 6.7 µM 5-FU dose of drug (Table 1) was combined with 1, 3, and 5 min of CAP. In five images, nuclei that were in focus were outlined and each mean fluorescence intensity (MFI) of Ki-67 channel was recorded. The mean of Ki-67 MFI was calculated for each treatment group including for No Treatment and Isotype control. A Ki-67^+^ cell threshold was determined as a cell with a MFI greater than the lowest mean of MFI of all groups other than Isotype control. There was a significant (cohort = 3, 2/cohort, n = 6, *t* test p < 0.05) decrease in cell count with FOLFIRINOX and 3 min of CAP treatment combined at 6 h compared to no treatment controls (Fig. 4A). In cells treated with combination CAP 3 min and FOLFIRINOX, less cells were observed (Fig. 4B,C). All cells were then graded as Ki-67^+^ or Ki-67^-^ on this scale. Representative images at the 3-min CAP timepoint and total cell counts of all timepoints are shown in Fig. 4. Ki-67 can be seen co-localized within the outlined nucleoli in cells regardless of treatment group (Fig. 4B,C).

### Induction of cell cycle arrest with combination treatment

**Figure 5:**
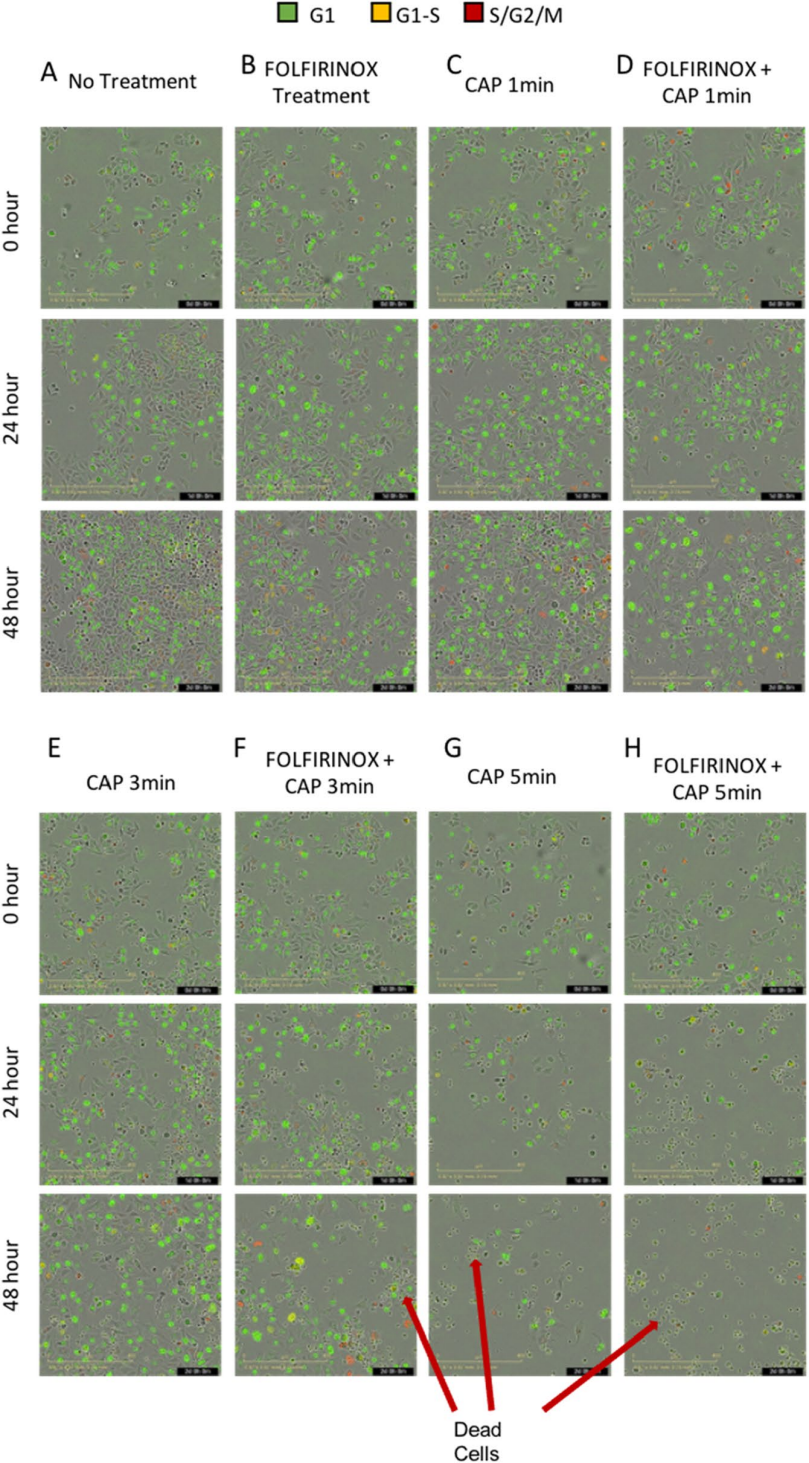
(**A**–**H**) Representative phase contrast images of fluorescently labeled KKU-055 cells 0, 24, and 48 h after no treatment, FOLFIRINOX (6.7 µM 5-FU, 1.7 µM leucovorin, 0.6 µM irinotecan, and 0.5 µM oxaliplatin), CAP at 120p (28.7 W) for 1, 3 or 5 min, and both in combination.

Experiments were designed to measure cell confluence and cell cycle distribution after combining the 6.7 µM 5-FU dose of FOLFIRINOX (Table 1) and CAP at 1, 3, and 5 min. Cells were placed in the IncuCyte Live Cell imaging system immediately after CAP where confluence was monitored. Representative images of 0 h, 24 h, and 48 h timepoints are shown to demonstrate cell confluence within treatment wells (Fig. 5A–H). In Fig. 5, morphological differences can be seen between experiment conditions. No treatment and drug only treated cells are confluent at 48 h with most cells visibly fluorescent. In combination treatment wells, cells are not confluent and large clusters of cellular debris are visible 48 h after treatment.

**Figure 6:**
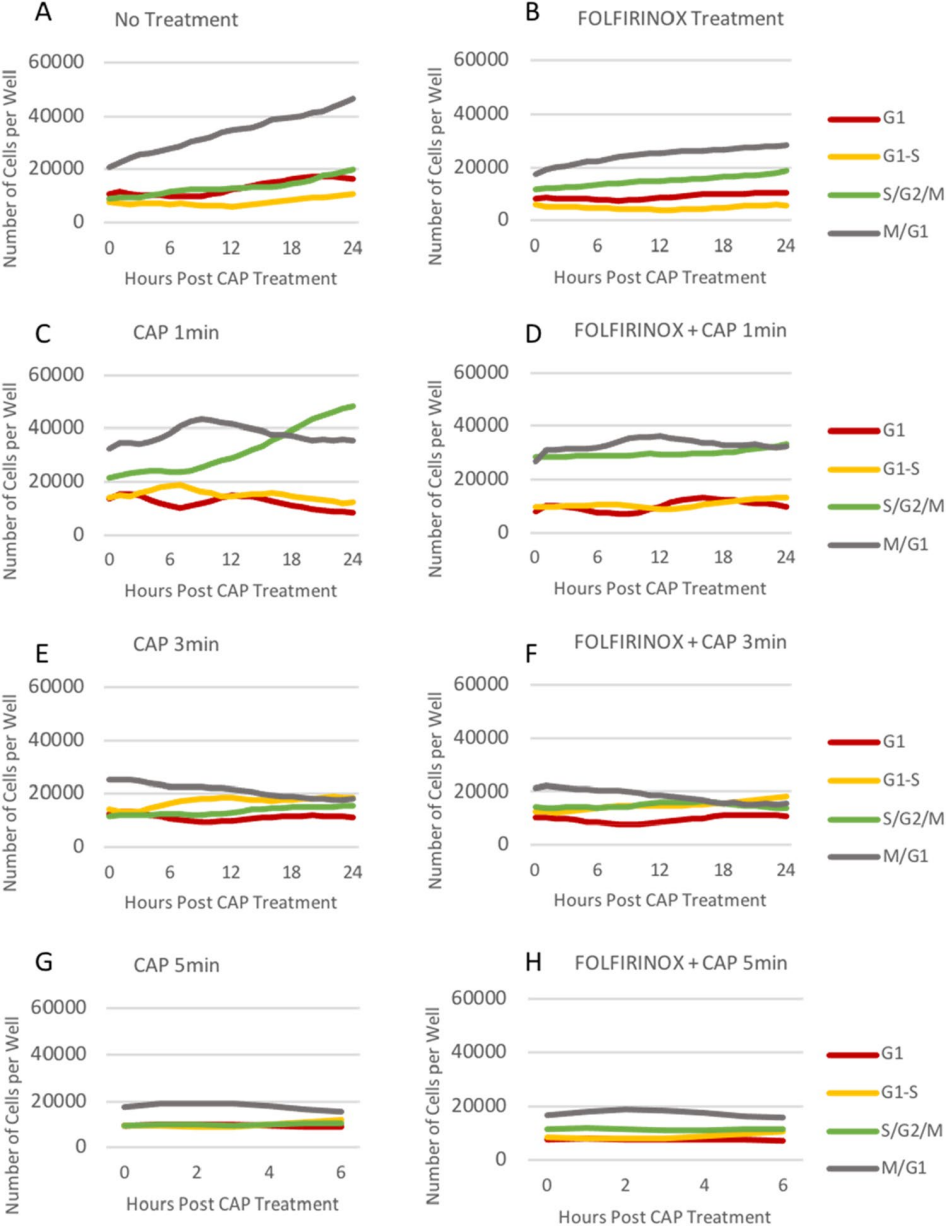
The FOLFIRINOX dosage of 6.7 µM 5-FU, 1.7 µM leucovorin, 0.6 µM irinotecan, and 0.5 µM oxaliplatin was combined with CAP at 1, 3, and 5 min to characterize the cell cycle response. (A-H) The number of cells in either G1 phase, G1-S transition, S/G2/M phase, or M-G1 transition per well in each treatment group from 0 to 24 or 0 to 6 h.

The number of cells in different phases of the cell cycle was quantified through fluorescence measurements. The quantifications of 0–24 h after CAP treatment at 1 and 3 min and 0–6 h after CAP for 5 min are plotted in Fig. 6A–H. In the no treatment and FOLFIRINOX only treated groups, most cells are in the mitotic phase (grey line), and the number of cells increases over time (Fig. 6A,B). Also, the number of cells in S/G2/M phase (green line) increases in these wells. Conversely, cells treated with FOLFIRINOX and CAP were not proliferating. At a CAP dosage of 1 min, cells were moving through the cell cycle, as shown in the cyclic lines for all phases (Fig. 6C). With a combination of FOLFIRINOX and CAP 1 min this progression is reduced, and the lines are less cyclic (Fig. 6D). At CAP dosages of 3 min, the grey line of cells in M-G1 phase trended down after treatment as apoptosis occurred (Fig. 6E,F). As shown in representative phase images Fig. 5E,F cells started to die at the 24 h timepoint. CAP and FOLFIRINOX combination treatment hindered the cell cycle, and the number of cells in the mitotic phase was reduced compared to FOLFIRINOX or a low dose (1 min) of CAP alone. At CAP dosages of 5 min, apoptosis initiated immediately after treatment and dead cells aggregated within 6 h (data not shown) therefore cell cycle data were not plotted beyond this timepoint in Fig. 6G,H. Cells treated with FOLFIRINOX and CAP in combination completely died within 24 h. Clusters of dead cells can be seen in Fig. 5G,H 24 h after CAP treatment.

## Discussion

Cholangiocarcinoma treatment research aims to improve available chemotherapeutic options and FOLFIRINOX is promising as a novel, effective, yet toxic treatment. A clinical goal now is to establish a standard FOLFIRINOX dosage based on clinical trials. Multiple phase 1 and 2 studies are underway with encouraging results for FOLFIRINOX treatment in different doses over gemcitabine plus cisplatin, however there is no standard. The early issues in these studies are toxicity of FOLFIRINOX and early triumphs show that the regimen can be safe in patients who are able to tolerate it. These trials attempt to minimize toxicities by reducing or modifying drug doses^11,33^.

CAP is a promising therapy for CCA because of its in vitro selectivity of bile duct cancer cells^25^. In a CCA mouse xenograft model, the application of CAP on the tumor did not produce systemic side effects, and was selective in the tumor microenvironment^25^. Systemic risks have not been extensively studied in human clinical cases due to limited CAP use on patients. The lack of severe side effects in humans has been documented in one cohort of 20 patients with oral cancer^34^.

Strategies for integrating CAP treatment and chemotherapy have been emerging through in vitro combination studies over the last few years. CAP has already been combined in vitro and in vivo with gemcitabine treatment, a standard option in CCA and pancreatic cancer regimens^28,35^. In malignant melanoma cells, 24-h pretreatment with oxaliplatin before 30 s of CAP treatment had an additive effect to toxicity^26^. Another report examined breast cancer cells incubated with drug loaded nanoparticles for 24 or 72 h before CAP and saw synergistic inhibition of cell growth compared to individual treatments alone^27^. Strategies for combination experiments in vitro are crucial to understanding the interactions between CAP and drug therapies. These reports support a combined anti-tumor effect, demonstrating that CAP has potential to increase anti-tumor effectiveness of current medicines.

In this study, CAP generated by the CCPCS was combined with a FOLFIRINOX regimen to treat cholangiocarcinoma cells in vitro as there exists a need to examine interactions between CAP and novel chemotherapeutics. This study demonstrates that both CAP and FOLFIRINOX individually and in combination effectively reduce cell viability suggesting that FOLFIRINOX dosage can be reduced if paired with CAP for the treatment of CCA. Synergy was seen through MTT assays at various doses of FOLFIRINOX and CAP (Table 2). Confocal microscopy and IncuCyte imaging demonstrated a decrease in cell counts and changes in cell morphology after treatment which was consistent with the reduction in viability shown in Fig. 2.

The underlying mechanism of these synergetic results remains unknown. FOLFIRINOX treatment in vivo includes administration of multiple doses that may not be completed due to toxicity resulting in a lower or incomplete regimen. Surgical resection for CCA may not be an option for late-stage diagnosis due to the high recurrence rate^3,4^. Combination treatment strategies could increase the efficacy of surgical options for patients. In the JCRI-ABTS phase I FDA Investigational Device Exemption Approval clinical trial (G190165/R001) of CCPCS^36^, CAP is sprayed across the tumor bed immediately following resection. Here in the tumor microenvironment, CAP would interact with residual tumor cells and normal tissue. In CCA cases where recurrence is a concern, CAP has the potential to target the remnant microscopic tumor. For other cancers with high recurrence, CAP may benefit current chemotherapy regimens. However, the synergy reported in this data is limited to an in vitro model. The efficacy of combination treatment could be altered by other factors in vivo.

This is the first study to investigate the synergistic interaction between CAP and FOLFIRINOX for the treatment of cholangiocarcinoma. Our finding of synergism between CAP and chemotherapeutics has great potential. CAP and FOLFIRINOX can be combined to reduce cholangiocarcinoma tumor cell viability and proliferation. We determined the dosage combinations in which viability reduction could be enhanced by adding 1–5 min of low temperature plasma to a low dose of FOLFIRINOX (6.7 µM fluorouracil, 1.7 µM leucovorin, 0.6 µM irinotecan, and 0.5 µM oxaliplatin). A combination therapy would be advantageous for patients where an intense FOLFIRINOX regimen is too aggressive, and this warrants further clinical research. We focused on the low doses of FOLFIRINOX to reduce overall chemotherapeutic burden in vitro as a model of lower toxicity in vivo. If a lower dose of FOLFIRINOX is administered, patients with low performance status can have more treatment options. Knowledge of the interactions between CAP and chemotherapeutics is of clinical value and can lead to more personalized medicine and a lower chemotherapy burden on patients in the future.

## Conclusion

The effectiveness of Canady Helios Cold atmospheric plasma in combination with a FOLFIRINOX regimen was explored. We found that a combination treatment can be significantly more effective than either CAP or FOLFIRINOX alone in reducing cholangiocarcinoma cell viability. We are the first to demonstrate the in vitro synergistic effect of a FOLFIRINOX treatment and CAP, and our data suggests CAP could be a possible adjuvant therapy for cholangiocarcinoma. It is important that CAP alone can selectively induce tumor cell death, however our results demonstrate that CAP can potentially reduce the dose of chemotherapeutic drugs needed for cancer patients. Future studies may examine the cellular pathways involved in these synergistic characteristics and identify the ideal dose of treatment that has the lowest feasible toxicity with the most productive outcome. This study provides insights for the clinical application of CAP for cholangiocarcinoma cancer treatment.

## Abbreviations

CAP: Cold atmospheric plasma
CCPCS: Canady cold plasma conversion system
